# Itching eyes after itching around the head

**DOI:** 10.3205/oc000136

**Published:** 2020-02-27

**Authors:** Laura Tabuenca Del Barrio, Marcos Mozo Cuadrado, Alicia Zubicoa Enériz, Iñigo Martínez de Espronceda Ezquerro

**Affiliations:** 1 Complejo Hospitalario de Navarra, Ophthalmology, Pamplona, Navarra, Spain; 2 Complejo Hospitalario de Navarra, Dermatology, Pamplona, Navarra, Spain

**Keywords:** Phthirus pubis, pediculosis, nits, louse, lice

## Abstract

**Objective:** To report the occurrence and management of an eyelashes infestation by Phthirus pubis.

**Methods:** A 27-year-old female presented with itching in her right eye and head after she had been traveling in southern Italy five days earlier. Visual acuity (VA) was 20/20 in both eyes.

Slit-lamp examination showed bilateral blepharitis. Moreover, mobile insects and eggs attached to the eyelashes were observed. A microbiological study was performed with a Phthirus pubis result. The patient was treated with mechanical dislodging.

**Results:** The resolution of the infection was carried out removing every insect and egg. A vaseline application twice daily for 7 days was necessary to stifle any nits that could remain.

**Conclusions:** The eyelashes pediculosis is frequently caused by Phthirus pubis. Only a minor percentage of the cases are due to Phthirus capitis, but the differential diagnosis is essential: there are Phthirus pubis pediculosis cases due to sexual abuse. A sexual history and screening for other sexually transmitted diseases is warranted.

## Introduction

Pediculosis is a disorder caused by one of three varieties of louse: Phthirus pubis, Phthirus capitis, and Phthirus corporis.

Pediculosis ciliaris or phthiriasis palpebrarum is frequently caused by Phthirus pubis, also known as the crab louse. Only a minor percentage of pediculosis ciliaris is caused by Phthirus capitis. Here, we present the clinical manifestations, diagnosis, and management of a Phthirus pubis eyelashes infestation.

## Case description

**Figure 1:**
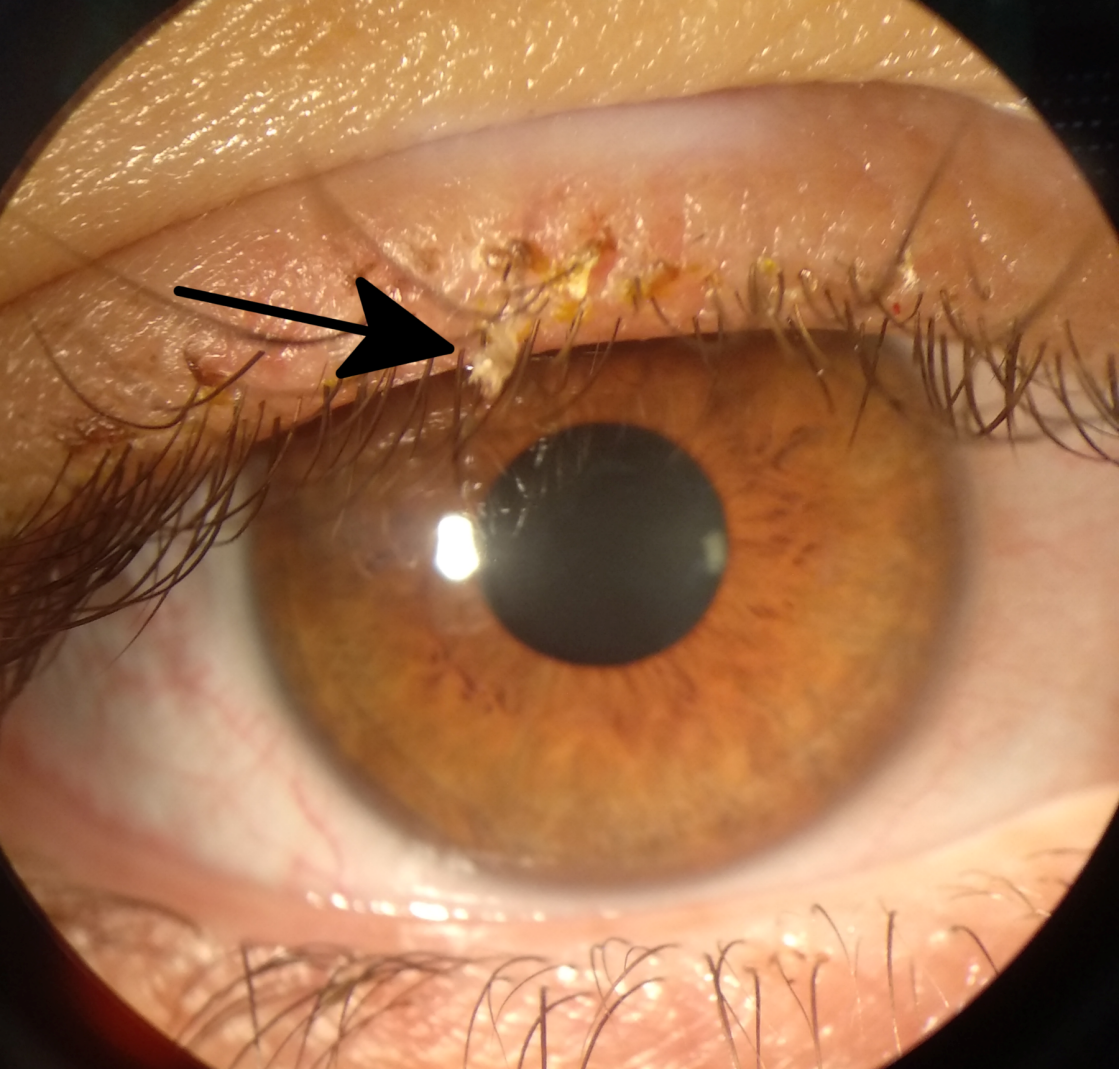
Slit-lamp photography; a translucent mobile Phthirus pubis hanging on the eyelash; blepharitis with scabs is observed too.

**Figure 2:**
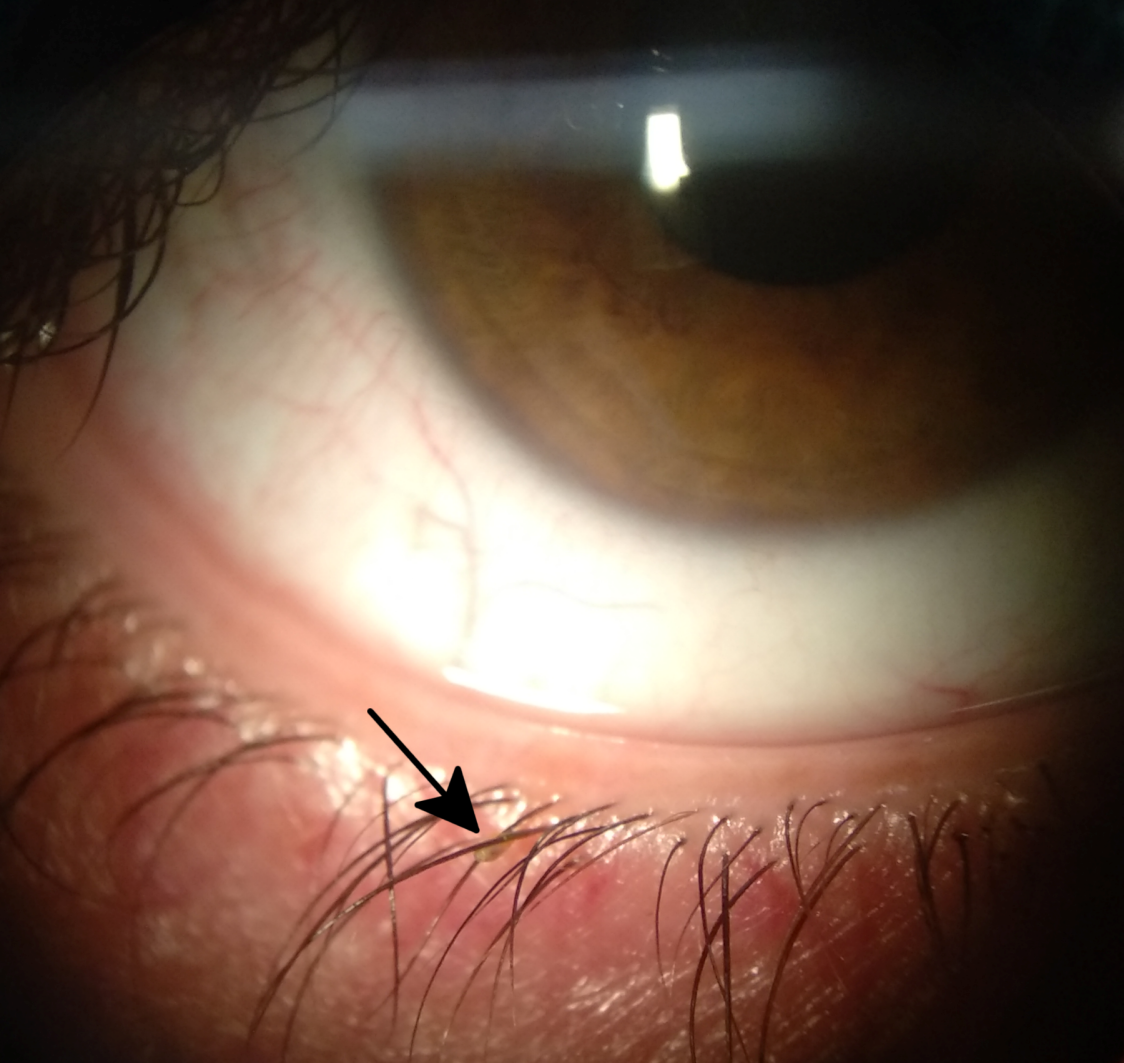
Slit-lamp photography; an egg with a nit inside is observed.

A 27-year-old female presented with itching in her right eye four days ago. As personal records, she had severe head itching after she had been traveling in southern Italy five days earlier. The patient thought that it might be a head lice infection and was treated with pyrethrins shampoo getting the head itch to disappear. Visual acuity (VA) was 20/20 (Snellen) in both eyes. Slit-lamp examination revealed bilateral blepharitis and some mobile insects attached to the eyelashes (Figure 1 (Fig. 1)). Looking carefully, translucent nits and eggs could be seen attached at the base of the eyelashes (Figure 2 (Fig. 2)). A mechanically dislodging was carried out removing every insect and egg. A microbiological study was performed with a Phthirus pubis result. The patient was diagnosed of pediculosis ciliaris. A vaseline application twice daily was recommended to asphyxiate any nit or louse that could remain.

## Discussion

The crab louse is a 1 mm long podgy parasite whose transparency makes it difficult to see unless filled with blood from a recent meal. It has four or six legs terminating in crab-like claws with which it holds to pubic or other body hairs. The average life of a female crab louse is three to four weeks and it lays about three eggs per day which hold firmly to the base of a hair. They incubate for one week before hatching [1].

Eyelashes infestation is usually presented in adolescents or adults with pediculosis pubis or children who have been in close contact with infested adults. It is frequently transmitted during sexual contact. Transmission via contact with fomites such as clothing, towels, or linen may also occur, but is thought to be less common [2]. We must question patients about the transmission. Even though the patients tell us that they have not had sexual contact, as happened in our case, we have to tell them that a screening for other sexually transmitted diseases should be done. If the patient is a child, pediculosis ciliaris may be a sign of sexual abuse and the possibility of abuse should be investigated [1].

Clinical manifestations are itching, burning, and eye irritation. The infection is frequently bilateral. Slit-lamp examination shows the eggs adhered to the eyelashes’ bases and mobile louses hanging from the eyelashes. If a careful exploration is not carried out, the infestation may be mistaken for blepharitis associated with seborrheic dermatitis, bacterial conjunctivitis, or allergic contact dermatitis [3]. The transparence of lice may generate confusion: they look like scabs.

In all cases, a microbiological study to confirm the diagnosis is necessary. Moreover, this also makes it easier for us to tell patients that a sexual study is required. Although some eyebrow and eyelashes Phthirus capitis clinical cases are described in patients with a great head infestation, it is very important to differentiate both parasites. Phthirus pubis is smaller with the body wider, the legs more asymmetrical and less mobile [4].

Treatment consists of direct removing with a clamp, looking through slit-lamp. Vaseline ointment is adequate to stifle the insects. There are no studies comparing the efficacy of different treatments for pediculosis ciliaris if mechanical dislodging and vaseline do not result productive [5]. The ocular irritation should be considered before employing any of the therapies raised such as pilocarpine gel (may involve a neurotoxic effect on lice), fluorescein drops, yellow mercuric oxide ointment, permethrin cream, or malathion shampoo.

## Conclusions

Phthirus pubis is the main cause of pediculosis ciliaris. A careful exploration is essential to differentiate the insects from the blepharitis scabs. A mechanical extraction is necessary to treat this infection. Vaseline application is an adjuvant agent that may facilitate the pathogen elimination. In all patients, a sexual history is required and a sexual transmission screening is recommended. In addition, its apparition in children requires suspicion of sexual abuse.

## Notes

### Competing interests

The authors declare that they have no competing interests.
